# Remote monitoring for heart failure management during COVID-19 pandemic

**DOI:** 10.1016/j.ijcha.2021.100724

**Published:** 2021-01-28

**Authors:** Enrico Bertagnin, Antonio Greco, Giuseppe Bottaro, Paolo Zappulla, Imma Romanazzi, Maria Daniela Russo, Marco Lo Presti, Noemi Valenti, Giuseppe Sollano, Valeria Calvi

**Affiliations:** Division of Cardiology, C.A.S.T., Azienda Ospedaliero-Universitaria Policlinico “G. Rodolico – San Marco”, University of Catania, Italy

**Keywords:** COVID-19, Cardiac Implantable Devices, Heart Failure, Physical Activity, Remote Monitoring, Telemedicine

## Abstract

**Background:**

COVID-19 pandemic impacted on heart failure patients’ lifestyle and quality of life, affecting both physical activity levels and state of health.

**Methods:**

Demographic data and device records were extracted for patients with heart failure in the 16 weeks at the turn of lockdown during pandemic. To explore the variability across the lockdown period, a week-to-week analysis was performed. Patients were interviewed to investigate physical activity and psychological insights. The primary endpoint was the variation in physical activity at the turn of lockdown.

**Results:**

At our facility, 2225 patients implanted with a cardiac device were screened and data were collected for 211 patients fulfilling the inclusion criteria. Patients’ physical activity significantly decreased in the lockdown period compared with the control period (active time per day 8.0% vs. 10.8%; relative reduction [RRR] 25.9%; p < 0.0001). A small decrease was noted for mean heart rate (70.1 vs. 71.7 beats per minute [bpm]; RRR 2.2%; p < 0.0001), while thoracic impedance slightly increased (82.2 vs. 82.7 ohm; RRR 0.6%; p = 0.001). Patients’ physical activity decreased from week 7 to week 11 (10.9% vs. 6.9%; RRR 36.7%; P < 0.0001) with an increase between week 11 and week 16 (6.9% vs. 8.5%; RRR 18.8%; P < 0.0001). Patients’ perceptions about physical activity showed a very low correlation with remote monitoring-assessed physical activity levels (r^2^ = 0.035, p = 0.039).

**Conclusions:**

Telemedicine and remote monitoring can explore the impact of COVID-19 pandemic on vital signs and physical activity levels of heart failure patients, playing a crucial role in the prediction of heart failure worsening during circumstances discouraging outpatient visits.

## Graphical abstract

**Figure:**
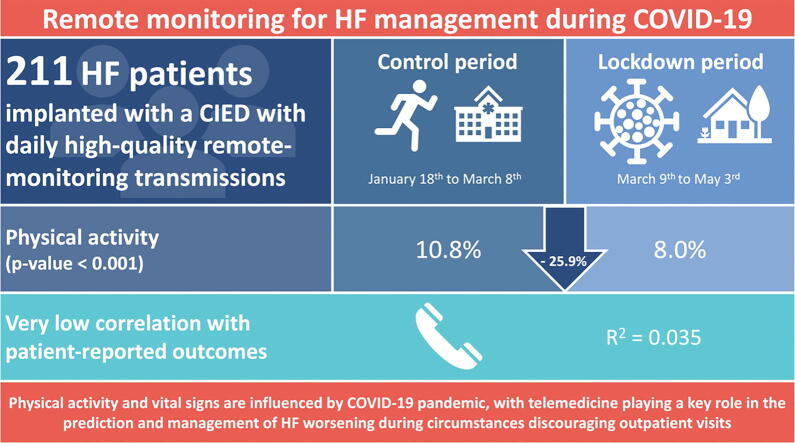

## 1 Introduction

Severe acute respiratory syndrome coronavirus 2 (SARS-CoV-2) pandemic has broken out, resulting into a global and rapid diffusion of the coronavirus disease 2019 (COVID-19), leading to excess mortality [1]. To limit viral spreading, governments issued restrictive measures, including forced homestay (i.e., lockdown), largely affecting people’s lifestyle and behaviors [2], [3]. Preliminary studies identified higher-risk subgroups, with patients suffering from heart conditions (e.g., heart failure [HF]) displaying a tendency towards worse outcomes, including severe or critical disease and mortality [4], [5].

Physical inactivity is acknowledged as a key modifiable risk factor for cardiovascular diseases and in particular for HF exacerbation [6]. In addition, beyond its detrimental effects on exercise, lockdown may increase anxiety and/or depression levels, which in turn are known to reduce physical activity in a chronic HF population [7]. An overview on physical activity reduction during the lockdown can be derived from patients implanted with a cardiac implantable electronic device (CIED). CIEDs are recommended for HF patients with severe systolic dysfunction to prevent sudden cardiac death (implantable cardioverter defibrillator, ICD) and/or to improve cardiac contractile function by resynchronizing left and right ventricles (cardiac resynchronization therapy, CRT) [8]. Advances in health technologies allow for an accurate remote monitoring of HF patients’ vital signs and activity indexes by collecting and forwarding data to the reference medical center [9]. This strategy is useful to early identify worsening HF patients and to optimize their pharmacotherapy to prevent decompensated HF recurrencies and hospitalizations [10], [11], [12].

Despite the need for human resources, telemedicine visits (phone call, videoconference, email) for HF patients during pandemic are proven to be effective in the reduction of adverse clinical outcomes [12]. Whether and to what extent HF patients’ physical activity varies as a response to lockdown and pandemic is still unclear. In addition, the feasibility of a remote monitoring-guided approach to provide medical support, reassurance and clinical benefits to HF patients is so far unexplored, now more than ever representing an unmet need.

The purposes of our study are as follows: firstly, to investigate the direct impact of COVID-19 pandemic on HF patients’ physical activity levels and vital signs; secondly, to determine the contribution of the indirect effect of the lockdown (i.e., anxiety and depression) on HF patients’ health status; thirdly, to explore whether telemedicine and remote monitoring can play a role in the management of chronic HF patients amidst circumstances discouraging outpatient visits.

## 2 Methods

### 2.1 Study population

Patients were considered eligible for the inclusion in our study if they met the study inclusion criteria: (1) Age ≥ 18 years old; (2) Chronic HF with reduced left ventricular ejection fraction (HFrEF); (3) Implantation with a CIED (ICD or CRT) and periodic outpatient follow-up at our facility; (4) Active Biotronik Home Monitoring (Biotronik SE&Co. KG, Berlin, Germany) providing daily high-quality data on electrical parameters, vital signs and physical activity levels.

To minimize heterogeneity and to select daily high-quality remote monitoring data on the variables of interest, the inclusion in the current study was limited to patients with active Home Monitoring. Patients with low remote monitoring transmission rates (defined as <20% of programmed transmissions) were excluded from our analysis.

### 2.2 Study design

Demographic data, clinical indication to CIED implantation and remote monitoring records were extracted from the Home Monitoring system and subsequently entered an interim database for data sorting and aggregation. To investigate the effects of lockdown and to detect potential variations across the study period, individual patient data from 16 weeks at the turn of the lockdown date were collected and categorized into two groups: (1) lockdown period (from March 9th to May 3rd, as of Italy government decree) [13]; (2) control period (from January 18th to March 8th, defined as a comparable timeframe before restrictive measures were issued). During the lockdown period, people could not leave their home except for serious reasons, including health problems, work purposes and necessities.

For the purposes of this analysis, individual patient data were aggregated and summarized to derive two central tendency measures for each variable (i.e., median values during the control and lockdown periods). These figures were finally collected into the final database for between-periods comparisons. To explore the variability of the parameters of interest within the lockdown period, a week-to-week analysis was performed, selecting three representative weeks for comparisons, i.e. week 7 (before lockdown, from February 24th to March 1st), week 11 (during lockdown, from March 23rd to March 29th) and week 16 (final lockdown, from April 27th to May 3rd).

Furthermore, to investigate the network among forced lockdown, physical activity variations and patients’ mindset, study participants were asked to undergo a phone-call interview, during which they were asked to rate their physical activity levels before lockdown (from 0 [complete inactivity] to 4 [athletic lifestyle]) and to disclose whether a decline (mild, moderate or severe) was noticed during the lockdown period. As part of the interview, study participants were also asked information about their compliance to cardiovascular pharmacotherapy and were administered two specific and broadly validated questionnaires (i.e., the Zung self-rating depression and anxiety scales) to assess their psychological attitude towards pandemic and lockdown [14].

### 2.3 Endpoint definitions

The primary endpoint of our study was the variation between control and lockdown periods in terms of physical activity levels. Physical activity was assessed by remote monitoring as the percentage time during which any type of exercise was detected with regards to the whole monitored daily time (24 hours).

Secondary outcomes of interest included the following: variation in the mean heart rate between control and lockdown periods; variation in thoracic impedance between control and lockdown periods; patients’ perceptions on physical activity across the lockdown period and their matching with observed modifications; increase in anxiety and/or depression levels during the lockdown, assessed by the Zung self-rating questionnaires [14].

### 2.4 Statistical analysis

The distribution of each variable of interest was explored using the Kolmogorov-Smirnov test. Continuous variables were summarized as means with standard deviation in case of normal distribution, or medians with interquartile range if the Kolmogorov-Smirnov test showed skewed data. To detect mean ranks differences among repeat measurements of non-Gaussian variables in a single sample (paired data), the Wilcoxon signed-rank test was used [15]. Similarly, to ensure the identification of differences across multiple test attempts, the week-to-week analysis availed from the Friedman test [16]. Correlation between variables of interest was explored with the Spearman's rank correlation test [17]. All p values were based on two-sided tests. A p value less than 0.05 was considered to be statistically significant for all analyses. For multiple comparisons, the Bonferroni correction was applied [18]. All the analyses were performed using the Statistical Package for Social Sciences (SPSS Inc., Chicago, Illinois), version 25.

## 3 Results

### 3.1 Study population

**Fig. 1:**
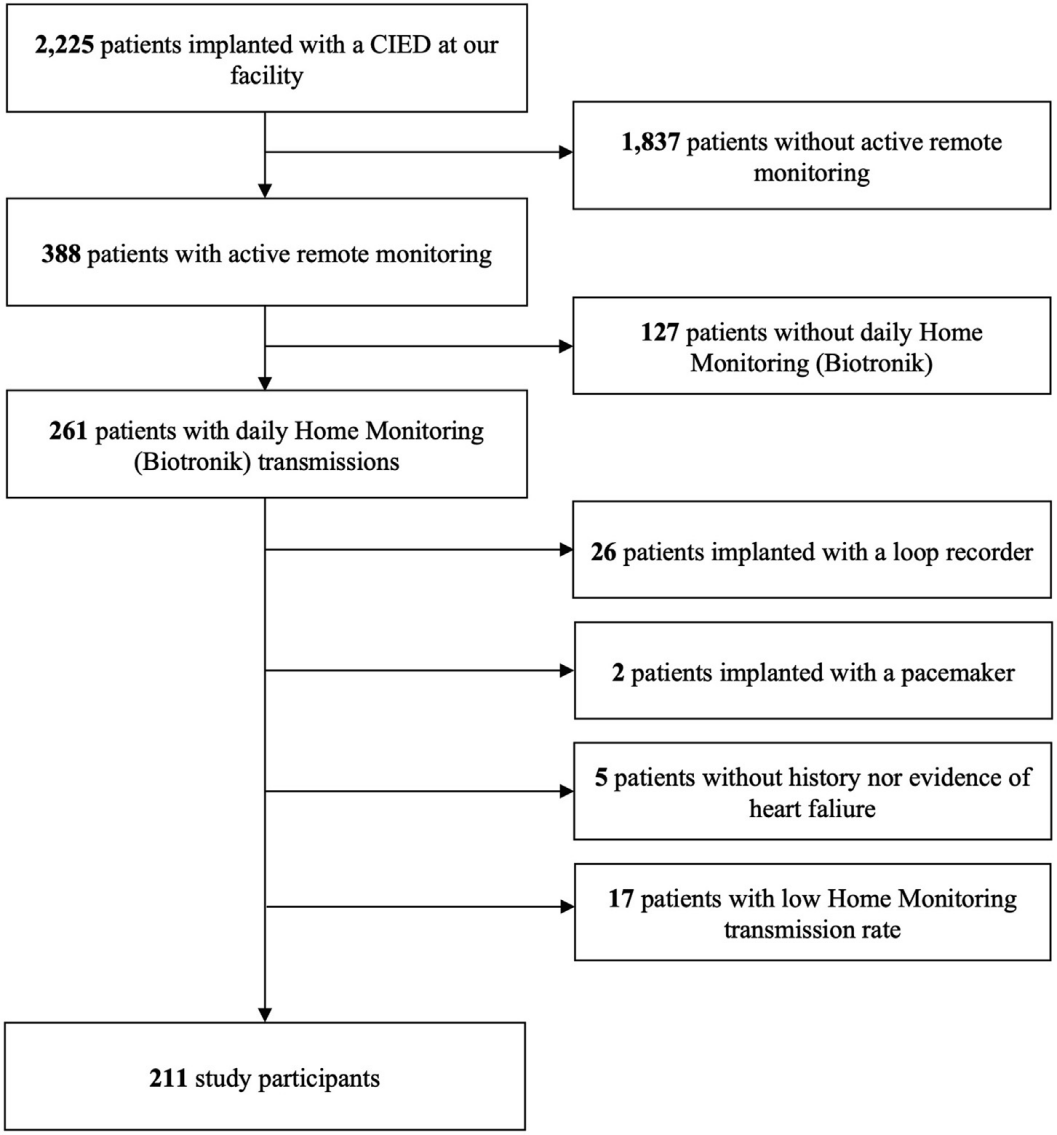
Study flow-chart. Abbreviation: CIED = Cardiac Implantable Electronic Device.

Patients’ screening and selection process was conducted as showed in the study flow-chart (Fig. 1). Overall 2225 consecutive patients implanted with a CIED and followed-up at Policlinico “G. Rodolico – S. Marco” (Catania, Italy) were initially screened for the inclusion in the current study. Patients without active remote monitoring were excluded, thus restricting our population to 388 subjects. To minimize heterogeneity and to select patients with daily high-quality data transmission, the inclusion in the current study was restricted to 261 patients with active 1Home Monitoring (Biotronik SE&Co. KG, Berlin, Germany). To keep consistency in device characteristics, remote monitoring parameters and clinical indication behind CIED implantation, we further excluded 2 patients implanted with a pacemaker, 26 with an implantable loop recorder and 5 with ICD or CRT for secondary prevention purposes (i.e., patients without HF diagnosis). In addition, 17 patients were excluded due to low remote monitoring transmission rate (defined as <20% of the programmed transmissions). As a result, 211 HF patients with daily high-quality remote monitoring data were deemed to be eligible for the inclusion in the current analysis, therefore representing the study population.

### 3.2 Baseline characteristics

Study participants displayed a mean age of 68.6 ± 9.5 years and were predominantly male (N = 161, 76.3%). A mean time of 36 ± 24 months has elapsed since device implantation.

With respect to CIED type, we can distinguish different subgroups: 86 patients (40.8%) were implanted with a CRT device, while 12 (5.7%) and 113 (53.5%) with a dual- and single-chamber ICD, respectively. Among single-chamber ICD patients, 28 patients (24.8%) have a ventricular lead with DX technology, therefore enabling atrial sensing through a floating dipole into the right atrium [19].

### 3.3 Physical activity and vital signs

**Fig. 2:**
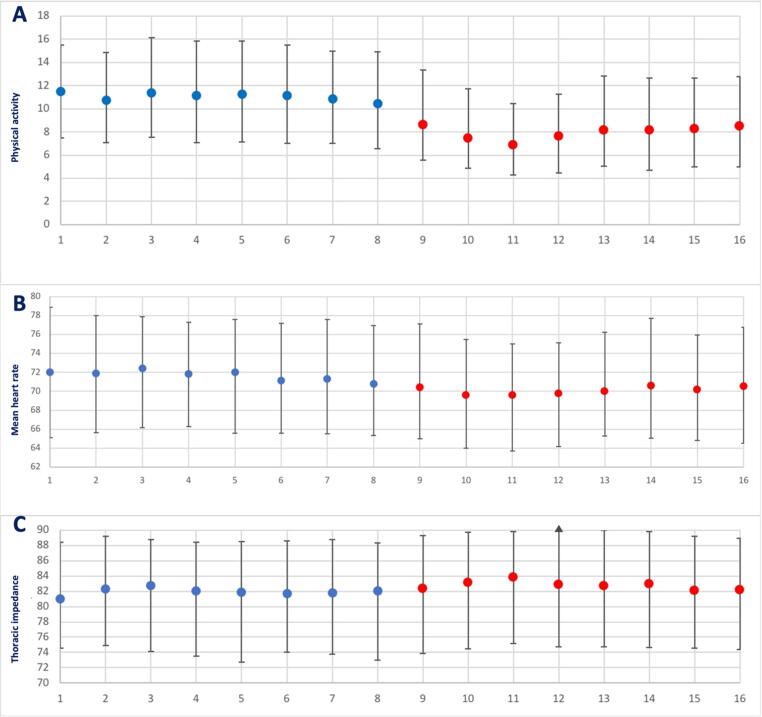
Physical activity and vital signs variations between control and lockdown periods. In the panel A, physical activity levels are reported through study weeks, while panel B shows the differences in mean heart rate, and the panel C refers to variations in thoracic impedance. Blue indicates control period, and red lockdown period. (For interpretation of the references to colour in this figure legend, the reader is referred to the web version of this article.)

Patients’ physical activity significantly decreased during the lockdown period as compared to the control period (active time per day 8.0% vs. 10.8%; relative risk reduction [RRR] 25.9%; p < 0.0001; Fig. 2, **panel A**). A small decrease was noted for mean heart rate (70.1 beats per minute [bpm] vs. 71.7 bpm; RRR 2.2%; p < 0.0001; Fig. 2**, panel B**) and mean resting heart rate (60.4 bpm vs. 61.7 bpm; RRR 2.1%; p < 0.0001), while thoracic impedance slightly increased (82.2 ohm vs. 82.7 ohm; RRR 0.6%; p = 0.001; Fig. 2**, panel C**). Interestingly, no difference was noted between control and lockdown periods in terms of ventricular tachyarrhythmias, antitachycardia pacing, shock therapies, or atrial fibrillation burden (Table 1).

**Table 1:** Remote monitoring-assessed vital parameters.

Variable	Control period	Lockdown period	p value
Mean percentage patient activity per day, median (IQR)	10.8 (6.9–15.2)	8.0 (5.1–12.7)	<0.0001
Mean heart rate, median (IQR)	71.7 (65.9–76.9)	70.1 (64.8–75.8)	<0.0001
Mean resting heart rate, median (IQR)	61.7 (56.8–67.2)	60.4 (56.6–65.8)	<0.0001
Thoracic impedance, median (IQR)	82.2 (74.0–88.3)	82.7 (74.6–89.8)	0.002
Atrial fibrillation burden, mean ± standard deviation	5.3 ± 22.0	5.4 ± 21.4	–
Ventricular tachycardia, number	76	67	–
Ventricular fibrillation, number	8	3	–
Antitachycardia pacing, number	74	37	–
Shock therapies, number	10	14	–

Statistical testing for arrhythmic events and device therapies was not possible due to the highly skewed distribution of the data.

*Abbreviations:* IQR, Interquartile range.

### 3.4 Week-to-week analysis

**Fig. 3:**
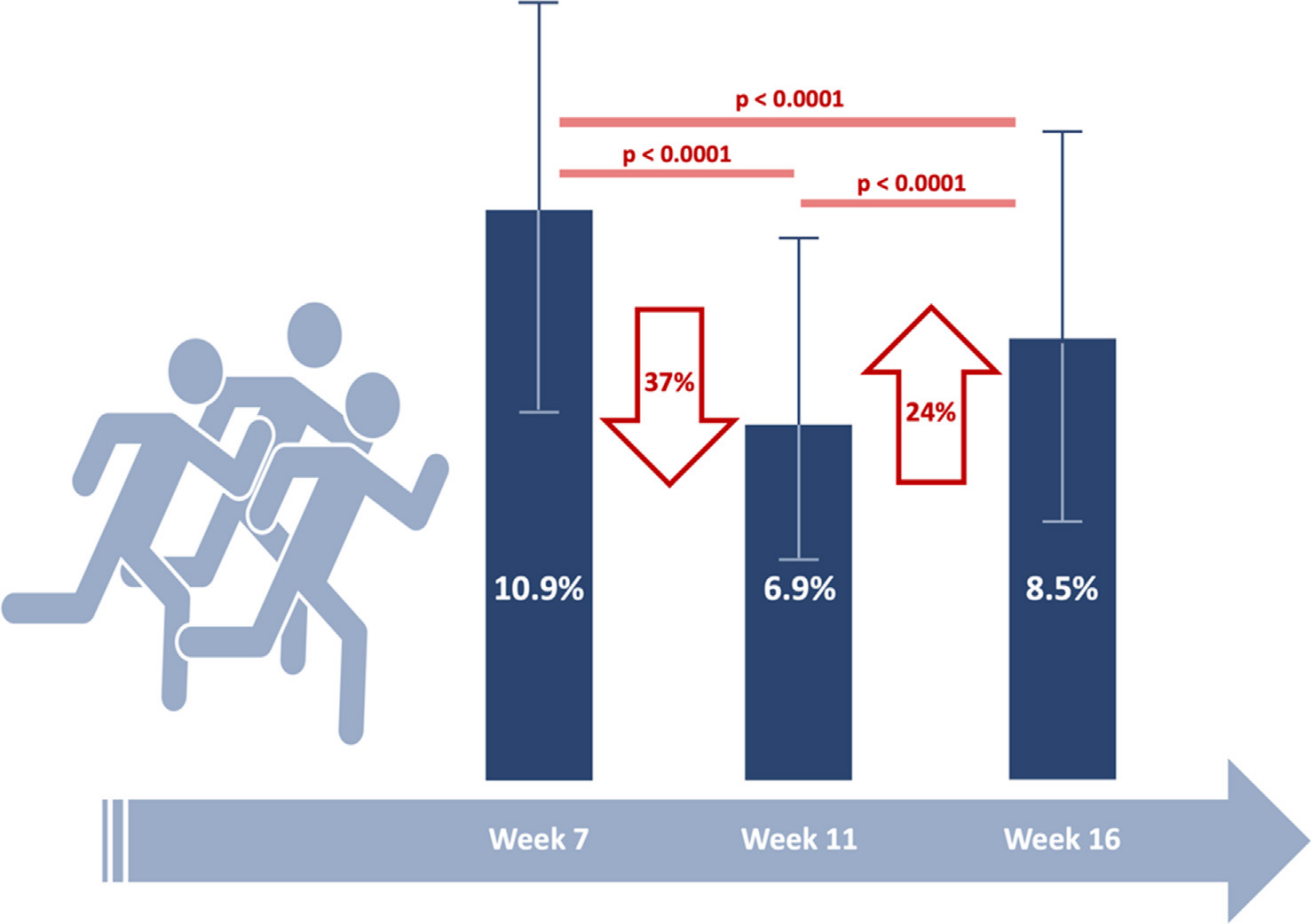
Week-to-week analysis of physical activity levels. Physical activity levels are compared between sample weeks (week 7 for control period, week 11 for the initial lockdown, week 16 for final lockdown). Red arrows indicate relative variations between comparisons. (For interpretation of the references to colour in this figure legend, the reader is referred to the web version of this article.)

In the week-to-week analysis, data from sample weeks 7, 11 and 16 were compared. Patients’ physical activity significantly decreased from week 7 to week 11 (10.9% vs. 6.9%; RRR 36.7%; P < 0.0001) with a subsequent slight increase between week 11 and week 16 (6.9% vs. 8.5%; RRR 18.8%; P < 0.0001; Fig. 3). Minor fluctuations across the lockdown period can be noted in mean heart rate (70.9 bpm, 68.9 and 70.3 at week 7,11 and 16, respectively) and thoracic impedance (81.7 ohm, 83.5 ohm and 81.8 ohm at week 7, 11 and 16, respectively).

### 3.5 Patient-reported outcomes

The phone-call interview was accepted and successfully completed by 125 (59.2%) study participants (Table 2). Two out of three included subjects (66.9%) reported a moderate-to-high physical activity level at baseline. In addition, a reduction in exercise levels during the lockdown was perceived by a large proportion of patients (63.1%). Notably, patients’ perceptions about physical activity showed a very low correlation with remote monitoring-assessed physical activity levels (r^2^ = 0.035, p = 0.039).

**Table 2:** Patient-reported outcomes on physical activity, medications and psychological status.

*Self-reported physical activity*	
Inactivity	2/124 (1.6%)
Minimum activity (daily routine only)	39/124 (31.5%)
Moderate activity (walk)	73/124 (58.9%)
High activity (running, gym)	10/124 (8.1%)
Very high activity (competitive sport)	0/124 (0%)

*Self-reported decrease in physical activity during lockdown period*	
No reduction	45/122 (36.9%)
Grade 1 (Minimum)	17/122 (13.9%)
Grade 2 (Mild)	20/122 (16.4%)
Grade 3 (Moderate)	22/122 (18.0%)
Grade 4 (High)	11/122 (9.0%)
Grade 5 (Very high)	7/122 (5.7%)

*Have you ever forgotten to take your medications?*	
No	88/124 (71.0%)
Yes	36/124 (29.0%)

*If yes, did it occur more frequently during the lockdown period?*	
No	34/36 (94.4%)
Yes	2/36 (5.6%)

*Did you tend to forget the same medication?*	
No	29/36 (80.6%)
Yes	7/36 (19.4%)

*Self-reported anxiety level*	
No anxiety	114/125 (91.2%)
Mild to moderate anxiety	11/125 (8.8%)
Marked to severe anxiety	0/125 (0%)
Extreme anxiety	0/125 (0%)

*Self-reported depression level*	
No depression	115/125 (92.0%)
Mildly depressed	9/125 (7.2%)
Moderately depressed	1/125 (0.8%)
Severely depressed	0/125 (0%)

Data are presented as numbers and percentages.

After the administration of the Zung self-rating questionnaire for anxiety, a mean value of 34.2 ± 7.8 was found (normal anxiety level). The majority of study participants (N = 114; 91.2%) reported normal anxiety levels, whereas 11 subjects (8.8%) scored for a level of mild anxiety; no patient disclosed moderate to high anxiety levels. No correlation was found between the anxiety score and variations in physical activity (evaluated as difference between control and lockdown periods), mean heart rate, mean resting heart rate and thoracic impedance.

Likewise, the median depression score by the Zung self-rating questionnaire for was 32.0 (interquartile range 28.0–38.5). While 115 participants (92.0%) scored in the normal range, 9 (7.2%) and 1 (0.8%) patient could be considered to suffer from mild or moderate depression, respectively. Severe depression was reported by none of the participants. Similar to anxiety, no correlation was found between depression levels and other study variables.

The vast majority of the study participants (71.0%) reported an optimal compliance to prescribed pharmacotherapy (i.e., no missed doses for any of the medications).

## 4 Discussion

The main findings of the current study are as follows. Firstly, patients’ physical activity levels significantly decreased during the lockdown period as compared to the control period. Secondly, small reductions in mean heart rate, mean resting heart rate and thoracic impedance were noted during the lockdown period. Thirdly, patients’ perceptions about physical activity poorly correlated with the remote monitoring-assessed physical activity levels. Finally, anxiety and depression levels did not correlate with patients’ physical activity.

### 4.1 Study implications

This study assessed main modifications occurred during the COVID-19 pandemic in a population of HF patients implanted with a CIED. Forced homestay and physical activity levels during the lockdown offered a peculiar overview of the acute effects that physical inactivity may exert in the real-life setting, playing as a potential risk factor for HF recurrencies and delay in the access to care. A recent survey of 1,047 subjects highlighted the impact of the lockdown on physical activity levels, with an estimated reduction of 24% [20]. Preliminary reports showed that hospitalizations due to decompensated HF significantly declined during the COVID‐19 pandemic, yet hospitalized patients displaying more severe symptoms at admission [21]. This may be surrounded by a significant delay in hospital admissions due to patients’ fear of contagion and crowded areas, as noted for other acute events [22]. Similarly, psychological factors influence the choice of long-term cardiovascular pharmacotherapy. Preliminary warnings arose about the safety of angiotensin converting enzyme inhibitors and angiotensin II receptor blockers with regards to the spreading of COVID-19. However, subsequent studies and their meta-analyses disavowed these concerns, supporting the recommendation for continuing these drugs by European and American cardiovascular societies [23].

Physical activity levels, mean heart rate, resting mean heart rate and thoracic impedance from CIED remote monitoring are broadly known to be helpful in predicting clinical outcomes in HF patients. Physical inactivity has been largely studied as a crucial risk factor in HF patients, with lower physical activity levels associated with a higher risk for recurrencies and hospitalization [24]. Interestingly, our study showed that the extent of physical activity reduction varied across the lockdown period, with a greater reduction across the first two weeks. Similarly to physical activity, mean heart rate plays a key role as a predictor of poor prognosis in HF patients [25], [26], [27]. Thoracic impedance has been used in the management of HF patients as well, acting as a proxy measure for pulmonary fluid status [28]. Indeed, it is well-known to be inversely correlated with pulmonary fluid balance and capillary pressures, with a reduction occurring before HF recurrencies, symptoms onset and requirement for hospital admission [29]. A routine monitoring of thoracic impedance may signal an upcoming fluid overload suggesting the potential for optimization of medical therapy [30]. The MOMOTARO II (Monitoring and Management of OptiVol Alert to Reduce Heart Failure Hospitalization II) was a prospective randomized trial investigating the role of thoracic impedance in patients with CIEDs to explore the role of lifestyle modification and pharmacological prevention of acute HF. In particular, patients assigned to the lifestyle modification group (reduction of sodium, water intake and daily activity for 1 week) showed an increase in thoracic impedance of 6.2% (from 63.3 ± 9.6 to 67.2 ± 10.1; P < 0.001), signaling a reduction of ﻿lung congestion. In our study, the percentage decrease of thoracic impedance was slightly lower than in the MOMOTARO II trial, probably due to the observational design of our anaylsis [31].

### 4.2 Study limitations

Our study should be interpreted in the light of several limitations. Firstly, this study represents a retrospective analysis exploring data extracted from CIED remote monitoring of only a manufacturer. Secondly, no active management of pharmacotherapy was performed based on the results of remote monitoring. Thirdly, 17 patients were not included in our analysis due to the low quality of remote monitoring transmissions; we cannot exclude differences in baseline characteristics of these patients, as a result of a selection bias. Finally, no clinical information was available for study participants due to COVID-19 pandemic discouraging outpatient follow-up visits.

## 5 Conclusions

Forced homestay during the COVID-19 pandemic played a key role in modifying vital signs and physical activity levels of HF patients, who tended to display poor insights about their physical activity, with a very low correlation degree between patient-reported and remote monitoring-assessed physical activity levels. Based on these findings, telemedicine and remote monitoring may be of crucial importance in the prediction of HF worsening during circumstances discouraging outpatient visits.

## Declaration of Competing Interest

The authors report no relationships that could be construed as a conflict of interest.
